# Improved Accuracy of High-Throughput Phenotyping From Unmanned Aerial Systems by Extracting Traits Directly From Orthorectified Images

**DOI:** 10.3389/fpls.2020.587093

**Published:** 2020-10-21

**Authors:** Xu Wang, Paula Silva, Nora M. Bello, Daljit Singh, Byron Evers, Suchismita Mondal, Francisco P. Espinosa, Ravi P. Singh, Jesse Poland

**Affiliations:** ^1^Department of Plant Pathology, Kansas State University, Manhattan, KS, United States; ^2^Interdepartmental Genetics, Kansas State University, Manhattan, KS, United States; ^3^Instituto Nacional de Investigación Agropecuaria (INIA), Programa de Cultivos de Secano, Estación Experimental La Estanzuela, Colonia del Sacramento, Uruguay; ^4^Department of Statistics, Kansas State University, Manhattan, KS, United States; ^5^Global Wheat Program, International Maize and Wheat Improvement Center, Mexico City, Mexico

**Keywords:** High-throughput phenotyping, unmanned aerial systems, canopy temperature, normalized difference vegetation index, ground cover, wheat

## Abstract

The development of high-throughput genotyping and phenotyping has provided access to many tools to accelerate plant breeding programs. Unmanned Aerial Systems (UAS)-based remote sensing is being broadly implemented for field-based high-throughput phenotyping due to its low cost and the capacity to rapidly cover large breeding populations. The Structure-from-Motion photogrammetry processes aerial images taken from multiple perspectives over a field to an orthomosaic photo of a complete field experiment, allowing spectral or morphological trait extraction from the canopy surface for each individual field plot. However, some phenotypic information observable in each raw aerial image seems to be lost to the orthomosaic photo, probably due to photogrammetry processes such as pixel merging and blending. To formally assess this, we introduced a set of image processing methods to extract phenotypes from orthorectified raw aerial images and compared them to the negative control of extracting the same traits from processed orthomosaic images. We predict that standard measures of accuracy in terms of the broad-sense heritability of the remote sensing spectral traits will be higher using the orthorectified photos than with the orthomosaic image. Using three case studies, we therefore compared the broad-sense heritability of phenotypes in wheat breeding nurseries including, (1) canopy temperature from thermal imaging, (2) canopy normalized difference vegetation index (NDVI), and (3) early-stage ground cover from multispectral imaging. We evaluated heritability estimates of these phenotypes extracted from multiple orthorectified aerial images via four statistical models and compared the results with heritability estimates of these phenotypes extracted from a single orthomosaic image. Our results indicate that extracting traits directly from multiple orthorectified aerial images yielded increased estimates of heritability for all three phenotypes through proper modeling, compared to estimation using traits extracted from the orthomosaic image. In summary, the image processing methods demonstrated in this study have the potential to improve the quality of the plant trait extracted from high-throughput imaging. This, in turn, can enable breeders to utilize phenomics technologies more effectively for improved selection.

## Introduction

In the past 20 years, spectacular advances in “next-generation” DNA sequencing have rapidly reduced the costs of genotyping and provided almost unlimited access to high-density genetic markers, thus allowing genetic improvement of several economically important crops worldwide (Crossa et al., 2017). Accurate plant trait (i.e., phenotypes) observations have long been the key to enhancing genetic gains through classical plant breeding (Eathington et al., 2007) and also to training prediction models and predict the performance of non-phenotyped individuals from their marker scores (Hayes and Goddard, 2001). Thus, phenotyping plays an essential role in the success of standard phenotypic selection as well as genomic selection models. Reflecting this, the lack of methods for rapid and accurate phenotyping on large sets of germplasm under field conditions remains a bottleneck to genomic selection and plant improvement (Cabrera-Bosquet et al., 2012; Araus and Cairns, 2014). High-throughput phenotyping (HTP) platforms are needed to measure plant traits non-invasively (Reynolds and Langridge, 2016), reduce the labor of manual phenotyping (Cabrera-Bosquet et al., 2012; Cobb et al., 2013), and measure multiple traits, plots, or both efficiently and simultaneously (Barker et al., 2016; Wang et al., 2018).

Unmanned Aerial Systems (UAS)-based remote sensing is being broadly implemented for field-based high-throughput phenotyping due to its low cost and the capacity to cover large field trials with thousands or tens-of-thousands of plots (Shi et al., 2016). Recently, multi-rotor UAS in various sizes have been widely deployed at a low altitude (<50 m) in HTP of plant canopy spectrum features (Haghighattalab et al., 2016; Li et al., 2018), plant growth status (Chu et al., 2017; Singh et al., 2019), and crop water use (Thorp et al., 2018). With the rapid development of low-cost consumer-grade sensors and platforms, UAS phenotyping holds great potential to be an integral part of plant genomics and breeding for precise, quantitative assessment of complex traits on large populations.

Structure-from-Motion (SfM) based photogrammetry is a process widely used to quantify plant phenotypes from aerial images (Shi et al., 2016). In SfM, a large number of aerial images taken from multiple perspectives over a field are used to create an orthomosaic image of a complete field experiment. Then plant traits can be extracted from a defined area (i.e., a shapefile of boundary coordinates for individual plots) within the orthomosaic image. However, during the generation of the orthomosaic image through SfM photogrammetry, pixels within the overlapped area from multiple raw images are blended. For instance, there are multiple optional blending modes such as mosaic and average for orthomosaic image generation in Agisoft Photoscan (Agisoft, 2018). The blending of pixel values has the potential to introduce changes in values intrinsic to the raw images.

Here, we use a complex plant trait – canopy temperature (CT) – as an example of a trait difficult to accurately quantify in the field environment. CT is an indicator of plant water stress and is often correlated with grain yield (Balota et al., 2007). Compared to measuring CT by sensors on the ground (Crain et al., 2016), using the aerial vehicles integrated with thermal imaging sensors can rapidly cover the observation area and potentially reduce diurnal temperature variations. Sagan et al. (2019) compared the performance of measuring CT in soybean and energy sorghum using three commercial thermal cameras on a UAS platform. They demonstrated a high correlation (*R*^2^ > 0.9) between temperature extracted from orthomosaic images and ground measurements at noon time assuming minor temperature changes in a short period of UAV-based imaging. By contrast, on much larger field trials with many thousands of entries, longer measurement windows (i.e., hours) often give raise to temperature fluctuations. In such cases, a single CT value extracted from an orthomosaic image may not accurately reflect the actual temperature at each imaging time point. Yet, variation in CT during the measurement window could be thoroughly characterized and accounted for by extracting thermal values directly from the series of raw images, rather than the single orthomosaic image.

An additional confounding factor for thermal imaging is more technical, specifically the flat field correction (FFC) from the thermal camera that may negatively impact the thermal image quality. The FFC consists of a re-calibration of the thermal camera core while the camera is working^1^. FFC is helpful to regulate the thermal data within a defined range of temperature readings but leads to continual recalibration and hence, yields varying values during image acquisition, including temperature differences from the same target in two consecutive images. The error may be compensated using the temperature references on the ground, but the referencing target may not be present in every image. This adds additional complexity to the field operations and image processing. If images with inaccurate thermal measurements are used for mosaicking, the CT values extracted from the orthomosaic image will likely reflect artifacts from both the FFC and the variability of thermal values from changing ambient conditions during the measurement window. To reduce this effect, Deery et al. (2016) proposed an approach to extract CT from images on a frame-by-frame basis. However, this method requires automation if it is to be used for processing data from large nurseries and for genetic studies with thousands or tens-of-thousands of plots.

Based on the issues described above, measurements of CT using thermal cameras are hypothesized to be substantially influenced by the SfM processing required for mosaicking. Meanwhile, other types of remote sensing datasets are expected to have similar issues from the SfM processing pipeline. For example, the canopy normalized difference vegetation index (NDVI) and the leaf ground cover are widely used remote-sensing traits associated with grain yield and agronomic traits (Rutkoski et al., 2016; Duan et al., 2017). During aerial image acquisition, the reflectance measurements from the canopy changes according to the camera position and the solar angle. This reflectance change can be accurately quantified using a goniometer system and the bidirectional reflectance distribution function (BRDF) under natural illumination conditions on the ground (Sandmeier and Itten, 1999). Also, the leaf area may appear in different densities depending on camera view angles at nadir or off-nadir. Therefore, a single orthomosaic image composed of blended pixels can be expected to have similar problems to reflect variability throughout the measurement window.

According to the theory of SfM processing and the issues previously described, the overall objective of this study is to enhance quality of trait extraction from field-based data collection and measurement using high-throughput phenotyping by UAS remote sensing. For this purpose, we developed a set of image processing methods to extract phenotypes from orthorectified aerial images. We then compared these extracted remote-sensed phenotypes with the same phenotypes extracted from orthomosaic images. In addition to evaluation of CT, we investigated applying this trait extraction method to NDVI and the early-stage ground cover (GC). We fitted four competing linear mixed models to each trait and compared the models using estimates of broad-sense heritability and the Bayesian Information Criterion. Broad-sense heritability is a measure of the proportion of phenotypic variance that is due to all genetic effects relative to unaccounted error variance (Holland et al., 2003). The main purpose of estimating heritability is to understand the level of genetic control of a given phenotype which directly relates to the expected gain from different selection strategies, which is the fundamental concept of plant breeding. A high heritability value is indicative of higher precision and less error and is also connected to higher predictive ability for a given trait (Crain et al., 2017). In comparing methods for analysis of a fixed dataset, a higher heritability reflects that a given method accounts for more variance through decreasing the experimental error. We illustrate the proposed approach using data from three wheat breeding nurseries planted at different locations and in different years as case studies, whereby each nursery provided data on one type of phenotype.

## Materials and Methods

### Plant Material and Field Layout

Spring wheat (*Triticum aestivum* L.) breeding lines used for CT measurements were from the International Maize and Wheat Improvement Center (CIMMYT) wheat breeding program. The trials were planted on November 21, 2017, at Norman E Borlaug Experiment Station (27°22′57.6″N, 109°55′34.7″W) in Ciudad Obregon, Sonora, Mexico during the 2017–18 season. The experiment consisted of 1800 unique spring wheat entries distributed in 60 trials. Each trial was arranged as an alpha lattice design in two blocks. Plots served as experimental units and were 1.7 m × 3.4 m in size, consisting of raised bed planting on two beds spaced 0.8 m apart with paired rows on each bed at 0.15 m spacing for each plot. Details are in the Supplementary Table 1.

Winter wheat (*Triticum aestivum*) breeding lines from Kansas State University wheat breeding program were used for canopy NDVI and early-stage ground cover measurements. One trial for canopy NDVI measurements was sown on September 19, 2017 at the KSU Ashland Bottom Agronomy Farm (39°7′54.2″N, 96°37′12.6″W), Manhattan, Kansas, and the other trial for early-stage ground cover measurements was sown on September 17, 2018 at the KSU farm (39°7′56.4″N, 96°37′10.1″W). A total of 146 and 150 winter wheat entries were planted during the 2017–18 and 2018–19 season, respectively. During each season, the entire field experiment was arranged in two blocks. The entries included breeding lines and check varieties. In each block, a breeding line was planted in a single plot, while the checks were planted multiple times. The experimental plot was an individual six-row plot with 20 cm (8″) row spacing with plot dimensions of 1.5 m × 2.4 m. Details of each field experiment are listed in the Supplementary Table 1.

To improve the geospatial accuracy of orthomosaic and orthorectified images, ground control points (G) consisting of bright white/reflective square markers were uniformly distributed in the field experiment before image acquisition and surveyed to cm-level resolution. The GCPs in Obregon, Mexico were surveyed using a Trimble R4 RTK (Trimble Inc., Sunnyvale, California, United States) Global Positioning System (GPS). The GCPs in Kansas were surveyed using the Precis BX305 Real-Time Kinematic (RTK) Global Navigation Satellite System (GNSS) unit (Tersus GNSS Inc., Shanghai, China).

### UAS, Sensors, and Image Acquisition

The UAS used for image acquisition was a DJI Matrice 100 (DJI, Shenzhen, China). The flight plans were created using Litchi Android App (VC Technology Ltd., United Kingdom) and CSIRO mission planner application^2^ for DJI Matrice100. Accordingly, the flight speed, the flight elevation above the ground, and the width between two parallel flight paths were adjusted based on the overlap rate and the camera field of view. Both cameras were automatically triggered with the onboard GNSS unit following a constant interval of distance traveled. A summary of flight settings is listed in the Supplementary Table 2.

To collect the thermal image from the spring wheat nurseries, a FLIR VUE Pro R thermal camera (FLIR Systems, United States) was carried by the DJI Matrice 100. Ten 0.25 m × 0.25 m square white metal sheets mounted on 0.50 m posts were used as GCPs. Two data collections were conducted between 11AM and 1PM on March 2 and March 19, 2018, during the grain filling stage. The aerial image overlap rate between two geospatially adjacent images was set to 80% both sequentially and laterally to ensure optimal orthomosaic photo stitching quality. Both flights were set at 60 m above ground level (AGL) at 5 m/s and could cover the 3600 breeding plots in around 16 min. To preserve the image pixel information, the FLIR camera was set to capture Radiometric JPEG (R-JPEG) images.

A MicaSense RedEdge-M multispectral camera (MicaSense Inc., United States) was used to collect winter wheat canopy images in both the 2017–18 and the 2018–19 seasons. White square tiles with a dimension of 0.30 m × 0.30 m were used as GCPs. Nine and four GCPs were placed and surveyed in the field during the 2017–18 and 2018–19 season, respectively. All UAS flights were conducted between 11AM to 2PM. A total of five UAS flights were made during the grain-filling stage in the 2017–18 season, and four UAS flights were made in the early Fall establishment period for 2018–19 season. Detailed flight dates are listed in the Supplementary Table 2. The aerial image overlap rate between two geospatially adjacent images was set to 80% both sequentially and laterally to ensure optimal orthomosaic photo stitching quality. All UAS flights were set at 20 m AGL at 2 m/s and could cover 360 (2017–18 season) and 336 (2018–19 season) plots in 14 and 11 min, respectively. To preserve the image pixel intensity, the MicaSense RedEdge-M camera was set to capture uncompressed TIFF images.

### Orthomosaic and Orthorectified Images Generation

**FIGURE 1:**
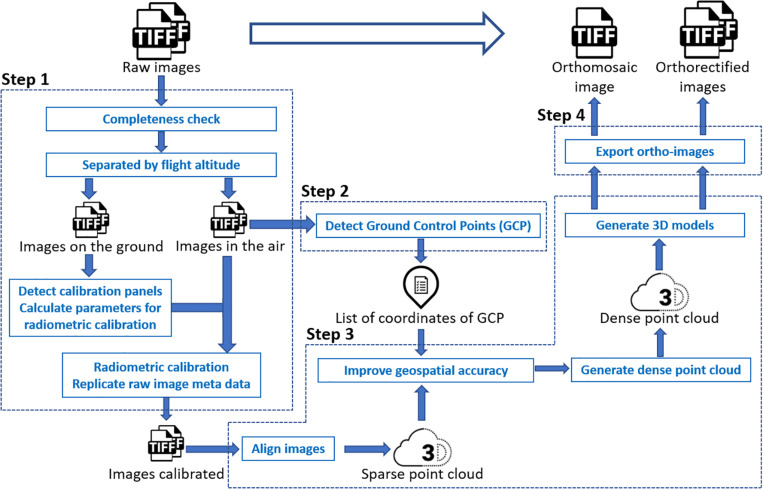
Workflow to generate orthomosaic and orthorectified images from raw images.

In this study, models fitted to a trait extracted from the orthomosaic image were used as a benchmark control, against which to compare estimates from models fitted to the same traits extracted from multiple individual orthorectified images. Unlike the approach proposed by Deery et al. (2016), in this study we still needed to generate the orthomosaic image of a complete field as a starting point to calculate the position of each individual image. Through the photogrammetry process, pixels in a raw image were projected to their real geographical location. Following this orthorectification, each individual raw image was converted to an orthorectified image. Therefore, there was no need to manually identify field plots in each orthorectified image because the same shapefile with plot boundaries could be used to identify a plot existing in different orthorectified images. Generating orthomosaic and orthorectified images from raw images consisted of (step 1) image preprocessing (including radiometric calibration), (step 2) GCPs detection, (step 3) photogrammetry process, (step 4) and export of orthomosaic image and orthorectified images (as shown in Figure 1), as explained below in detail. The procedure was implemented using Python, and the source code is available online^3^.

The image preprocessing procedure for the multispectral images converted the pixel value in each raw spectral image to reflectance before the photogrammetry process. Pixel values in raw thermal images, however, were not converted to temperature values in this step. As each trigger of the MicaSense RedEdge-M camera generated five images of every single spectral band (Blue, Red, Green, Near-infrared, and RedEdge), the completeness check removed images having less than five bands. According to the altitude (i.e., the camera height above the mean sea level) embedded in the image properties, images were divided into two groups – images captured on the ground and images captured in the air. The MicaSense radiometric calibration panels were then automatically detected from images captured on the ground if existing. Following the MicaSense radiometric calibration procedure^4^, calibration factors of all five bands were calculated and then applied to images captured in the air, converting raw images to reflectance images for subsequent photogrammetry process.

The GCPs detection procedure automatically identified the GCP in each image captured in the air if existing and matched the GCP with the surveyed position of the closest GCP from the image position. As white square tiles with the pre-known size were used as GCPs in the wheat field, clear patterns of GCPs could be detected through image processing. According to the image position (i.e., the longitude and latitude) embedded in the image properties, the surveyed GCP, whose coordinates were geographically close to the image position, was matched with the detected GCP in the image. Sufficient space (i.e., > 20 m) was left between every two GCPs during placement in the field to avoid having multiple GCPs in a single image and to enable sufficiently accurate geolocation of the UAS to determine which GCP was being imaged. All image file names and detected GCP coordinates were saved in a list for geospatial optimization in the photogrammetry process. Due to the low resolution of the thermal camera and the unclear pattern of GCPs in thermal images, GCPs were manually detected during the photogrammetry process of thermal images.

The photogrammetric processing of aerial images included sparse point cloud generation, geospatial optimization, dense point cloud generation, and 3-dimensional (3D) model generation. The process was implemented using the Agisoft PhotoScan Python API (Version 1.4.0, Agisoft LLC, Russia). An orthomosaic image of a complete field experiment was exported after the process. All images used to generate the orthomosaic image were exported as orthorectified images with the image boundary (i.e., the northwest and southeast corners) coordinates and the original camera position (i.e., longitude, latitude, and altitude) where the image was captured embedded in the image properties.

### Plot-Level Traits Extraction

**FIGURE 2:**
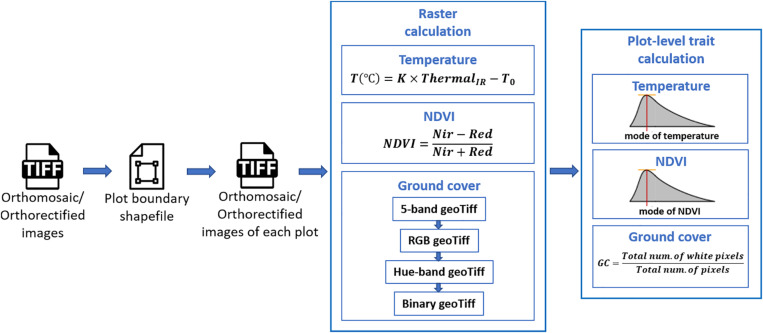
Workflow for plot-level trait extraction from orthomosaic and orthorectified images.

Extraction of plot-level phenotypic values from orthomosaic and orthorectified images consisted of (1) cropping single-plot images from an orthomosaic of the complete field or from multiple orthorectified images, each of which covered a small portion of the entire field, (2) converting pixel values to trait values through raster calculation, and (3) summarizing the plot-level trait in each image (as shown in Figure 2). The procedure was implemented using Python, and the source code is available online^5^.

Following the generation of the orthomosaic image of an entire field, a field map – a shapefile of polygons delineating the four corners of each plot was generated semi-automatically in Quantum Geographic Information System (QGIS, www.qgis.org) with the HTP Geoprocessor plugin (Wang et al., 2016). Specifically, the four corner points of the entire experiment field were first manually defined in QGIS. Then the coordinates of the four corners of the polygon for each plot were automatically calculated with the pre-known plot geometric size (length and width) using a QGIS Python script. Finally, each plot polygon was assigned a plot ID using the HTP Geoprocessor plugin (Wang et al., 2016). According to the field map, an image of each plot could be cropped from the orthomosaic image of the entire field experiment and saved as a GeoTiff image. Unlike the orthomosaic image of the complete field experiment, each orthorectified image only covered a small portion of the entire field. Therefore, only the plots that were completely included in the orthorectified image were cropped and saved as GeoTiff images. As a result, each plot was represented by a single cropped orthomosaic GeoTiff image and multiple orthorectified GeoTiff images.

To extract the CT trait, the pixel values within each GeoTiff image containing the thermal infrared band were directly used as indicators of absolute temperature measurements, as (1) the R-JPEG images have temperature data embedded in each pixel^6^ and (2) Sagan et al. (2019) has demonstrated the absolute temperature can be converted from the pixel value following a linear equation:

(1) $$T{(}^{\circ }\text{C})=K\times Thermal_{I\,R}-T_{0}$$

where *Thermal*_*IR*_ is the pixel value within the thermal infrared band of the GeoTiff image, *T* is the absolute temperature measurement in Celsius degrees, and *K* and *T*_0_ are constant parameters. In this study, *K* and *T*_0_ were set as 0.04 and −273.15 (Flir Systems Inc., 2017; Williamson et al., 2019; Song and Park, 2020).

To generate the NDVI trait from the GeoTiff image from the five-band multispectral GeoTiff image, the following equation was used during raster calculation:

(2) $$N\,D\,V\,I=\frac{N\,I\,R-R\,e\,d}{N\,I\,R+R\,e\,d}$$

where *NIR* and *Red* are the near-infrared and red band of the multispectral GeoTiff images, respectively, and *NDVI* is the output raster layer.

For the canopy GC calculation, the five-band multispectral GeoTiff image was first converted to an RGB GeoTiff image by rendering the Red, Green, and Blue bands. Then the RGB image was converted to a Hue-Saturation-Value (HSV) GeoTiff image. Finally, a binary image was generated from the Hue band of the HSV image by manually selected threshold values leaving white pixels representing the canopy area in the RGB image. In this study, the threshold value was selected from the first image data set (October 3, 2018) and was applied to the subsequent image data sets.

For extraction of CT and NDVI traits, we used the mode of all non-zero values (Figure 2) in a plot area as the plot-level CT and NDVI, respectively. This was intended to compensate for noise from the non-vegetative pixels within the plot area, although most of the plots were fully covered by canopies during image acquisition. The plot-level early-stage ground cover (GC in Figure 2) was calculated as the overall percentage of white pixels within the binary image. As a result, each type of plot-level trait extracted from the orthomosaic image had only one observation per plot, whereas the same traits extracted from orthorectified images had multiple observations, one per orthorectified image in which that given plot appeared complete.

Orthomosaic and orthorectified images collected on two dates (Supplementary Table 2), were used to extract two independent datasets for the CT trait. Similarly, images collected on five and four dates were used to extract five and four independent datasets for the NDVI and the GC traits, respectively (Supplementary Table 2).

### Statistical Analysis

Four general linear mixed models (models I to IV) were specified and fitted to each of the traits extracted, namely CT, NDVI and GC. For each trait, a null model (Model I) was fitted to observations extracted from the orthomosaic image (one observation per plot; Model I_*a*_) and to the average of the multiple observations per plot, as extracted from the orthorectified images (Model I_*b*_). The remaining three models (II, III, and IV) were fitted to the traits extracted from orthorectified images (i.e., multiple observations per plot) and were intended to recognize different aspects of the data collection process. Model fitting was implemented using the ASReml-R (Ver. 4) package in R (Butler et al., 2009; Gilmour et al., 2015), with variance components estimated by residual maximum likelihood (REML) (Butler et al., 2009; Gilmour et al., 2015). Additional details for each model follow.

### Model I

Model I was developed to fit a single observation per plot, with this single observation being either extracted from a single orthomosaic image per plot (*y*_*m*_, Model I_*a*_) or by averaging multiple plot-level observations ($y_{\bar{r}})$ extracted from orthorectified images (Model I_*b*_). Specifically,

(3) Model I:aym,i⁢j⁢k⁢l=μ(Ia)+Gi(Ia)+Bj(Ia)+Rk⁢(j)(Ia)+Cl⁢(j)(Ia)+ei⁢j⁢k⁢l(Ia)

(4) Model I:byr¯,i⁢j⁢k⁢l=μ(Ib)+Gi(Ib)+Bj(Ib)+Rk⁢(j)(Ib)+Cl⁢(j)(Ib)+ei⁢j⁢k⁢l(Ib)

where superscripts (I_*a*_) and (I_*b*_) indicate the model that each parameter corresponds to. Within each model, μ represents the intercept, *G*_*i*_ is the random effect of the i^th^ entry assumed distributed as iid $G_{i}∼N\,\left(0,\sigma_{G}^{2}\right),B_{j}$ is the random effect of the j^th^ block assumed distributed as iid $B_{j}∼N\,\left(0,\sigma_{B}^{2}\right),R_{k\,\left(j\right)}$ is the random effect of the k^th^ row nested within a block and assumed distributed as iid $R_{\left(j\right)\,k}∼N\,\left(0,\sigma_{R}^{2}\right),C_{l\,\left(j\right)}$ is the random effect of the l^th^ column nested within block and distributed as iid $C_{\left(j\right)\,l}∼N\,\left(0,\sigma_{C}^{2}\right)$ Finally, $e_{i\,j\,k\,l}^{\left({\text{I}}_{\text{a}}\right)}∼N\,\left(0,\sigma_{e^{\left({\text{I}}_{\text{a}}\right)}}^{2}\right)$ and $e_{i\,j\,k\,l}^{\left({\text{I}}_{\text{b}}\right)}∼N\,\left(0,\sigma_{e^{\left({\text{I}}_{\text{b}}\right)}}^{2}\right)$ are model-specific left-over residuals unique to the ijkl^th^ plot.

### Model II

Given the multiple observations on each plot that were extracted from orthorectified images (*y*_*r*_), it is possible to assess the variability between observations within a plot (i.e., within-plot variance) by expanding Model I as follows.

(5) $$\text{Model II}:y_{r,i\,j\,k\,l\,m}=\mu^{\left(\text{II}\right)}+G_{i}^{\left(\text{II}\right)}+B_{j}^{\left(\text{II}\right)}+R_{k\,\left(j\right)}^{\left(\text{II}\right)}+C_{l\,\left(j\right)}^{\left(\text{II}\right)}+\left(R\times C\right)\,{\left(B\right)}_{k\,l\,\left(j\right)}^{\left(\text{II}\right)}+\varepsilon_{i\,j\,k\,l\,m}^{\left(\text{II}\right)}$$

where μ, *G*_*i*_,*B*_*j*_,*R*_*k*(*j*)_, and *C*_*l(j)*_ are defined as for Model I. Meanwhile, (*R*×*C*)(*B*)_*kl*(*j*)_^(II)^ is the random effect of an individual plot identified by the combination of the k^th^ row and the l^th^ column within the j^th^ block, assumed iid distributed $\left(R\times C\right)\,{\left(B\right)}_{k\,l\,\left(j\right)}^{\left(\text{II}\right)}∼N\,\left(0,\sigma_{R\times C\,{\left(B\right)}^{\left(\text{II}\right)}}^{2}\right)$, and $\varepsilon_{i\,j\,k\,l\,m}^{\left(\text{II}\right)}$ is the leftover residual noise of the observation collected on the m^th^ orthorectified image of the ijkl^th^ plot, and assumed as iid distributed $\varepsilon_{i\,j\,k\,l\,m}^{\left(\text{II}\right)}∼N\,\left(0,\sigma_{\varepsilon^{\left(\text{II}\right)}}^{2}\right)$ Notably, in Model II, left-over residual terms $\varepsilon_{i\,j\,k\,l\,m}^{\left(\text{II}\right)}$ are unique to each ijklm^th^ observation within a given plot and thus represent technical replication (i.e., subsampling) of plots in the data collection process.

### Model III

**FIGURE 3:**
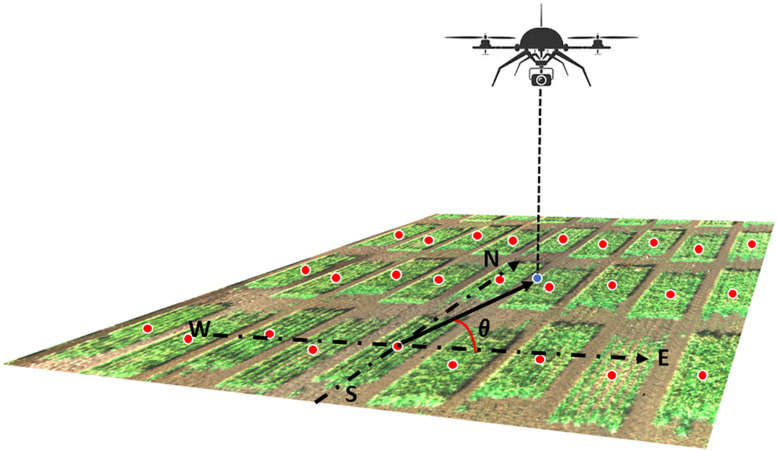
Illustration of the camera azimuth angle. The RGB image was captured by the UAS showing a small part of the field. The blue dot represented the camera projected position on the ground. Red dots represented the center of each plot. The camera azimuth angle (θ) was the angle between the true east (as 0°) and the vector from the plot center to the camera position.

**FIGURE 4:**
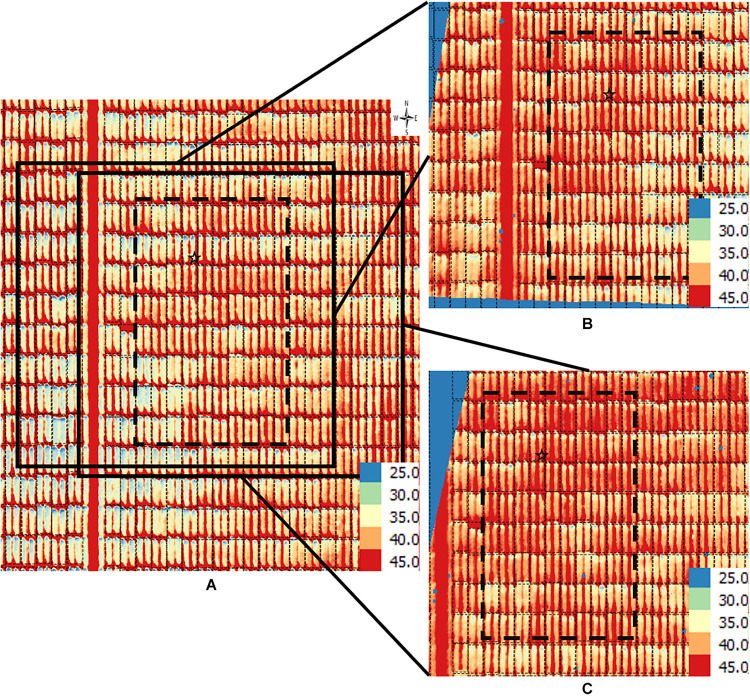
Orthomosaic and orthorectified images of CT. Raw thermal images for CT were captured on March 2, 2018, at 60 m AGL and were processed to generate **(A)** an orthomosaic image of the partial field and multiple orthorectified images of sections of the field, two of which are depicted here **(B,C)**. Black polygons delimited by thin dotted lines within each image delineate plot boundaries. Black polygons in thick dashed lines highlight a field section of interest common to the three images. In each image, a black star marks the same plot. The range of temperature (in Celsius degree) is marked in each image. The continuous blue areas **(B,C)** are non-effective pixels due to orthorectification to the raw images.

**FIGURE 5:**
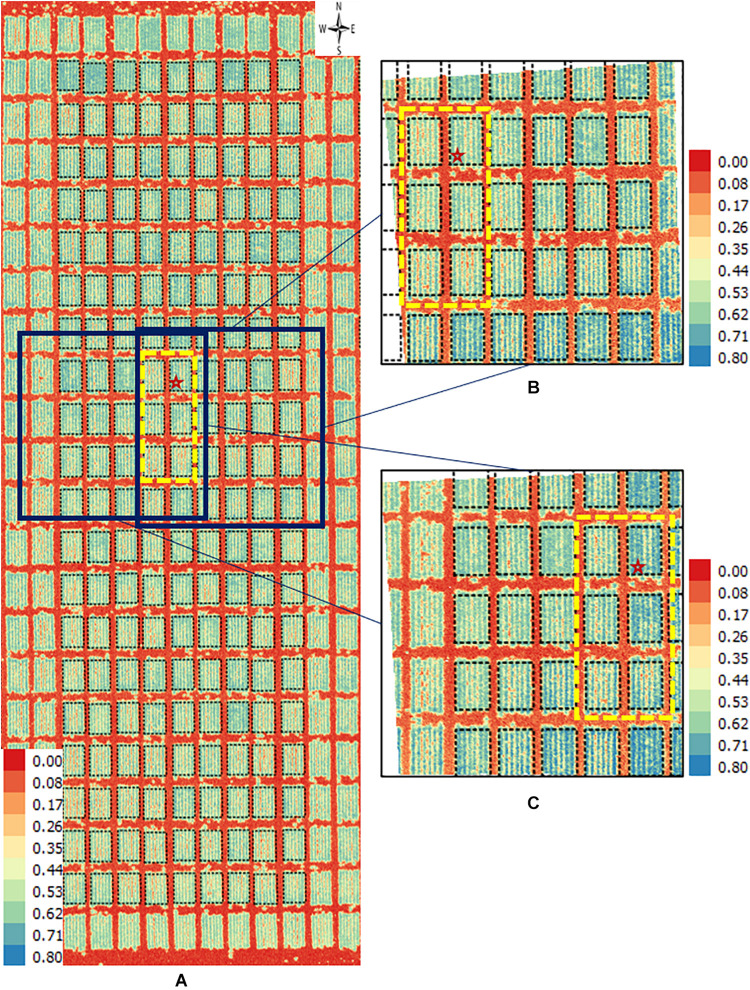
Orthomosaic and orthorectified images of NDVI. Raw images for NDVI were captured on April 4, 2018, at 20 m AGL from the 2017–18 wheat field experiment and were processed to generate **(A)** an orthomosaic image of a block in the field and multiple orthorectified images of sections of such block, two of which are depicted here **(B,C)**. Black polygons delimited by thin dotted lines within each image delineate plot boundaries. Yellow rectangles in dashed lines delimit the same subset of six plots in all three images. The range of NDVI (unitless) is marked in each image. The continuous white areas **(B,C)** are non-effective pixels due to orthorectification to the raw images.

Recall that each orthorectified image includes multiple plots in the camera field of view (Figures 3–5) and that images were captured by the UAS following a serpentine trajectory (moving along the column direction and turning around at the boundary rows) to cover the entire field. Therefore, for Model III, we consider replacing the specification of spatial effects of row and column with a clustering effect of image, as follows:

(6) $$y_{r,i\,j\,n}=\mu^{\left(\text{III}\right)}+G_{i}^{\left(\text{III}\right)}+B_{j}^{\left(\text{III}\right)}+I_{n}^{\left(\text{III}\right)}+G\times B_{i\,j}^{\left(\text{III}\right)}+\varepsilon_{i\,j\,n}^{\left(\text{III}\right)}$$

where μ, *G*_*i*_, and *B*_*j*_ are defined as for Model II. In turn, $I_{n}^{\left(\text{III}\right)}$ is the random effect of the n^th^ image and is assumed distributed as iid $I_{n}^{\left(\text{III}\right)}∼N\,\left(0,\sigma_{I^{\left(\text{III}\right)}}^{2}\right)$ Meanwhile, each plot is identified by the combination of the i^th^ entry in the j^th^ block, namely ${\left(G\times B\right)}_{i\,j}^{\left(\text{III}\right)}$ and assumed iid ${\left(G\times B\right)}_{i\,j}^{\left(\text{III}\right)}∼N\,\left(0,\sigma_{G\times B^{\left(\text{III}\right)}}^{2}\right)$ Finally, $\varepsilon_{i\,j\,n}^{\left(\text{III}\right)}$ is the left-over residual noise of the observation collected on the n^th^ orthorectified image of the ij^th^ plot, assumed distributed as iid $\varepsilon_{i\,j\,n}^{\left(\text{III}\right)}∼N\,\left(0,\sigma_{\varepsilon^{\left(\text{III}\right)}}^{2}\right)$ Much like in Model II, residual terms $\varepsilon_{i\,j\,n}^{\left(\text{III}\right)}$ in model III are unique to an observation within a plot and thus represent technical replication (i.e., subsampling) in the data collection process.

### Model IV

Model IV extends Model III to recognize that orthorectified images on a given plot are captured from different angles. Thus, Model IV incorporated camera view angle as an explanatory covariate in the linear predictor. This angle is defined from the center of the field plot to the camera’s position where the image is captured. As the UAS’s altitude could not be held constant during image acquisition, the absolute camera height above the ground level could not be accurately measured. Therefore, only the latitude and longitude (i.e., y and x coordinates) values of both the plot center and the camera were used to calculate the camera azimuth angle (Figure 3). Model IV was specified as follows:

(7) $$y_{r,i\,j\,n}=\mu^{\left(\text{IV}\right)}+X_{i\,j\,n}\,\beta^{\left(\text{IV}\right)}+G_{i}^{\left(\text{IV}\right)}+B_{j}^{\left(\text{IV}\right)}+I_{n}^{\left(\text{IV}\right)}+G\times B_{i\,j}^{\left(\text{IV}\right)}+\varepsilon_{i\,j\,n}^{\left(\text{IV}\right)}$$

where *X*_*ijn*_ is the camera azimuth angle corresponding to the n^th^ orthorectified image for the ij^th^ plot, β is the associated partial regression coefficient, and all remaining terms are defined as in Equation (6).

### Model Comparison

Specific model comparisons were targeted to address questions of interest. Specifically, Model I_*b*_ was compared to Model I_*a*_ to evaluate the effect of an averaged plot-level observation extracted from multiple orthorectified images compared to a single observation extracted from blended pixels in an orthomosaic image. Next, Model II was compared to Model I to investigate the effect of subsampling on estimation of the additive genetic variance (and functions thereof) based on multiple plot-level observations extracted from orthorectified images (II) compared to a single plot-level observation extracted from an orthomosaic image (I_*a*_) or from the average of multiple orthorectified images (I_*b*_). Furthermore, a comparison between Models II and III were intended to consider alternative ways of accounting for spatial variation, namely through rows and columns (II) vs. image clusters (III). Finally, Model IV expanded Model III to adjust for potential technical effects of the UAS with respect to the camera view angle.

Two metrics were selected for model comparisons, specifically the broad-sense heritability (*H*^2^) or repeatability, and the Bayesian Information Criterion (BIC) (Neath and Cavanaugh, 2012).

For all models, variance component estimates were used to compute *H*^2^ as follows. Specifically, to Models I_*a*_ and I_*b*_ (Equations 3 to 4), *H*^2^ was calculated as,

(8) $$H^{2}=\frac{\sigma_{G}^{2}}{\sigma_{G}^{2}+\frac{\sigma_{e}^{2}}{r}}$$

Using estimates of the entry-level variance $\sigma_{\varepsilon }^{2}$ and the plot-level variance $\sigma_{e}^{2}$ from Models I_*a*_ and I_*b*_, and *r* defined as the number of plots per entry (i.e., number of blocks). For Models II, III, and IV (Equation 5 to 7), the calculation of *H*^2^ included plot-level variance estimates (i.e., $\sigma_{R\times C\,{\left(B\right)}^{\left(\text{II}\right)}}^{2}$, $\sigma_{G\times B^{\left(\text{III}\right)}}^{2}$, $\sigma_{G\times B^{\left(\text{IV}\right)}}^{2})$, and estimates of $\sigma_{\varepsilon }^{2}$ characterizing subsampling, weighted by the number of subsamples (n) per plot, calculated as the harmonic mean number of observations across plots. Specifically, for model II

(9) $$H^{2}=\frac{\sigma_{G}^{2}}{\sigma_{G}^{2}+\frac{\sigma_{R\times C\,\left(B\right)}^{2}}{r}+\frac{\sigma_{\varepsilon }^{2}}{r\,n}}$$

And for each of Models III and IV:

(10) $$H^{2}=\frac{\sigma_{G}^{2}}{\sigma_{G}^{2}+\frac{\sigma_{G\times B}^{2}}{r}+\frac{\sigma_{\varepsilon }^{2}}{r\,n}}$$

As Models I_*a*_, I_*b*_, and II have different response variables, and BIC is used for model comparison assuming the same set of observations on the response variable, BIC is only used for Models II, III and IV in this study. Values of BIC were obtained from the ASReml-R (Ver. 4) package output. Smaller values of BIC are considered to indicate better fitting models.

### Data Availability

Data associated with these experiments, including the cropped, plot-level orthomosaic images and corresponding orthorectified images, can be accessed at the public repository^7^.

## Results and Discussion

### Orthomosaic and Orthorectified Image Generation and Gross Description

**FIGURE 6:**
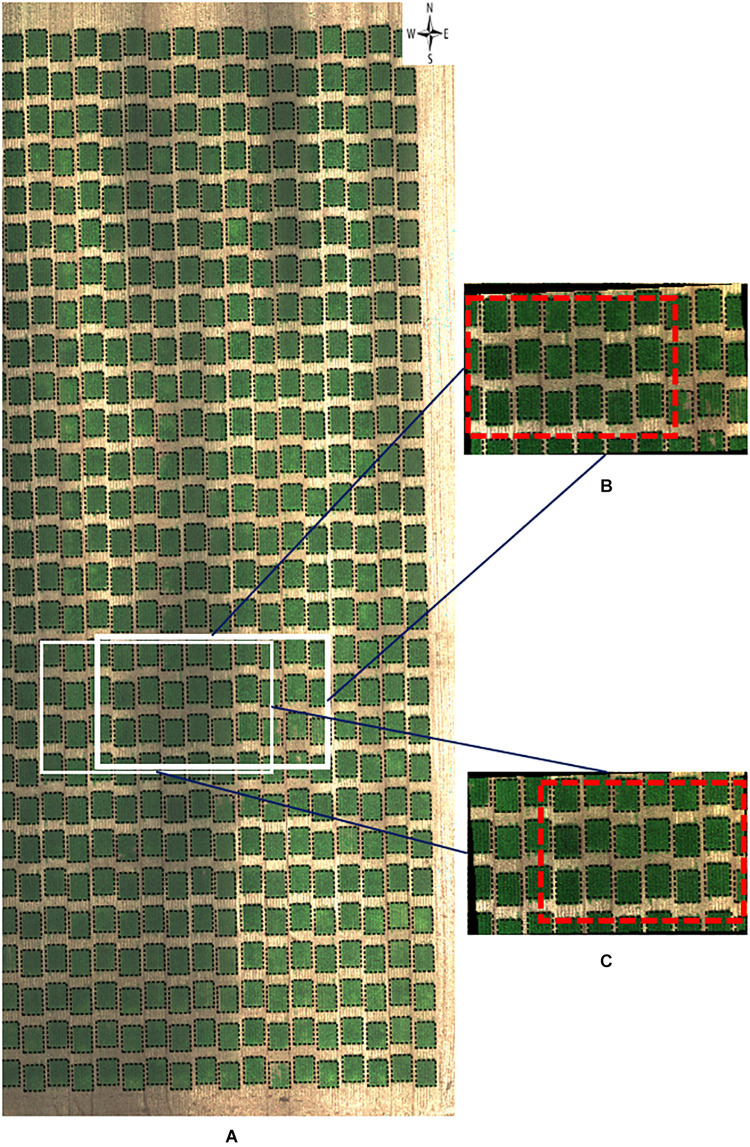
RGB orthomosaic and orthorectified images used for ground cover. Raw images were captured on November 3, 2018, at 20 m AGL from the 2018–19 wheat field experiment and were processed to generate **(A)** an RGB orthomosaic image of two blocks of the entire field, **(B,C)** two orthorectified sample RGB images illustrating different parts of the field. Black polygons in dashed lines within each image delineated plot boundaries. Red rectangles in dashed lines represented overlapped areas between two orthorectified RGB images. The continuous black areas **(B,C)** are non-effective pixels due to orthorectification to the raw images.

Using a case-study approach, we illustrate differences in image generation, gross description and corresponding trait extraction from orthomosaic images and orthorectified images. Specifically, for CT we used the March 2, 2018 dataset from spring wheat field (Figure 4), for NDVI we used the April 4, 2018 data from the winter wheat field (Figure 5), and for GC we used the November 3, 2018 data from the winter wheat field (Figure 6).

For the CT trait, gross differences in trait extraction are directly observable in a side-by-side comparison of the orthomosaic image with two of the orthorectified images (Figures 4A vs. 4B,C). Notably, all three images in Figure 3 show ranges in CT from 25°C (blue pixels) to 45°C (red pixels), as shown in the corresponding scales. Consider the individual plot marked with a star; the orthomosaic image seems to indicate a relatively low plot-level CT, based on more yellow pixels for that plot (Figure 4A). In contrast, the two orthorectified images show relatively high CT for the said star-marked plot, based on more orange and red pixels (Figures 4B,C). As observed, plot-level CT observations extracted from orthorectified images can disagree with the information available from the orthomosaic image, although not all orthorectified images reveal huge difference from the orthomosaic image on a given plot.

Similarly, directly observable differences were apparent between an orthomosaic and two orthorectified images for NDVI in wheat plots (Figure 5). Values of NDVI range from 0 (red pixels) to 0.8 (blue pixels) in all three images (Figure 5). For instance, consider the subset of six plots inside the yellow dashed rectangle in each of the three images. Although the difference is subtle, it is still visually detectable that more blue pixels in one orthorectified image (Figure 5C) than the other (Figure 5B). This perceived difference could be due to variation in reflectance over time due to the change of solar angle and different camera view angles. Another possible explanation may be digital artifacts of the camera, as it seemed that plots located at the east and south sides of both orthorectified images (Figures 5B,C) showed higher NDVI (i.e., more blue pixels) than plots in the remaining area of each image.

As for visual inspection of images of GC, we could not detect obvious GC differences between the orthomosaic image (Figure 6A) and the orthorectified images (Figures 6B,C). As GC is sensitive to the view angle from the camera to the plot, this observation supports that both ortho images (either orthomosaic or orthorectified) have been processed to apply corrections in the position of ground pixels caused by the perspective of the camera view angle. However, it is still unclear if pixel intensity may vary between orthomosaic and orthorectified images.

Taken together, the case studies presented here support potential variation in traits extracted from orthomosaic and orthorectified images. The main interest of a breeder is to quantify the genetic component of such variation; yet other sources of variation need to be considered and accounted for as well, namely environment factors (e.g., spatial effects) and imaging patterns due to the technology used for data collection (i.e., camera view angles and digital processing artifacts). Specifically, the illustrations presented above raised questions about the information contained in plot-level orthomosaic images generated by the photogrammetry process, as it was perceived to fail to accurately reflect trait variation that was directly apparent on plot-level observations originated from orthorectified images. Our concern is that the blending of pixel information that underlies the photogrammetry generation of orthomosaic images could lead to loss of information, thus undermining the quality of phenotypic data.

### Plot-Level Traits Extraction

Plot-level observations on CT, NDVI, and GC traits from two, five, and four datasets, each corresponding to a different data collection date (Supplementary Table 2), were extracted from orthomosaic and orthorectified images. For each trait, the total number of observations extracted from orthorectified images and the minimum, maximum, and median number of observations per plot extracted from orthorectified images were summarized (Supplementary Tables 3–5). The number of observations per plot extracted from orthorectified images ranged from 3 to 49 across traits. From an experimental design standpoint, each individual plot was assigned to a given genetic line. Thus, the multiple observations per plot extracted from the orthorectified images may be considered subsamples (i.e., technical replication) for the entry that plot was assigned to. By contrast, only one observation per plot was obtained for each trait from the orthomosaic image for a given field.

### Model Comparison

Table 1 shows estimated *H*^2^ for models I_*a*_, I_*b*_, II, III, and IV fitted to each of the traits extracted, namely CT, NDVI and GC. Table 2 shows BIC for model comparison between models II, III, and IV fitted to each of the traits extracted, namely CT, NDVI, and GC.

**TABLE 1:** Estimated broad-sense heritability (*H*^2^) for models I_*a*_, I_*b*_, II, III, and IV fitted to plot-level CT, NDVI, and GC observations on the case studies considered.

Date	Model I_*a*_	Model I_*b*_	Model II	Model III	Model IV
	**CT**				
**3/2/2018**	0.838	0.815	0.839	0.736	0.717
**3/19/2018**	0.606	0.825	0.834	0.615	0.619
	**NDVI**				
**4/4/2018**	0.432	0.572	0.598	0.348	0.389
**4/12/2018**	0.529	0.579	0.590	0.492	0.492
**4/19/2018**	0.262	0.633	0.696	0.311	0.370
**4/23/2018**	0.489	0.605	0.650	0.250	0.307
**5/16/2018**	0.489	0.399	0.422	0.370	0.467
	**GC**				
**10/3/2018**	0.811	0.794	0.799	0.843	0.877
**10/11/2018**	0.824	0.808	0.809	0.942	0.942
**10/21/2018**	0.706	0.700	0.700	0.878	0.882
**11/3/2018**	0.502	0.417	0.419	0.727	0.731

**TABLE 2:** Bayesian Information Criterion (BIC) for models II, III, and IV fitted to plot-level CT, NDVI, and GC observations on the case studies considered.

Date	Model II	Model III	Model IV
	**CT**		
**3/2/2018**	739726	620273	597315
**3/19/2018**	522193	512660	509143
	**NDVI**		
**4/4/2018**	−25028	−24832	−25417
**4/12/2018**	−31071	−30699	−30735
**4/19/2018**	−20666	−20804	−21751
**4/23/2018**	−23435	−23156	−24090
**5/16/2018**	−20888	−19871	−20777
	**GC**		
**10/3/2018**	−22314	−23872	−24529
**10/11/2018**	−19322	−15933	−15939
**10/21/2018**	−23489	−18529	−18552
**11/3/2018**	−22831	−20139	−20166

### Models I_*a*_, I_*b*_, and II

For both CT and NDVI, the magnitude of *H*^2^ estimates for Model I_*b*_ and Model I_*a*_ showed an inconsistent pattern across datasets (Table 1), though estimates seemed to be numerically greater in magnitude more often under Model I_*b*_. In contrast, for the GC trait, estimates of *H*^2^ were consistently greater in numerical magnitude under Model I_*a*_ than I_*b*_ based on the four datasets considered.

Comparing *H*^2^ estimates of all three traits between using Model I_*a*_ and Model II, we observed that *H*^2^ estimates of the CT trait on both two dates were improved by the latter model, as well as all *H*^2^ estimates of the NDVI trait except the one on the last date (Table 1). Taken together, for CT and NDVI traits, fitting multiple observations per plot into a hierarchical model that recognizes subsampling can help recover additive genetic variability in the data, as indicated by greater estimates of broad-sense heritability.

Compared to Model I_*b*_, Model II explicitly accommodated technical replication in phenotypic information, causing beneficial *H*^2^ estimates in all cases; however, the magnitude of gains ranged from moderate to marginal.

In summary, fitting phenotypic values of some crop traits (e.g., CT and NDVI) extracted from orthorectified images could increase estimates of *H*^2^ in some cases, relative to the same phenotypic traits obtained from orthomosaic images (Model I_*a*_). However, the estimation of *H*^2^ through fitting the GC trait could not be improved by simply replacing the single observation from the orthomosaic image with the average observations extracted from multiple orthorectified images. This inconsistent pattern was possibly due to the “mosaic” blending mode selected during photogrammetry processing of the orthomosaic photos. According to the Agisoft User Manual (2018), pixels were not simply blended by averaging pixel values from different photos in this blending mode, but through a pixel frequency related selection – a two-step approach. It was likely to be the reason why Model I_*b*_ and II could not always improve the trait estimation compared to Model I_*a*_. Further research is required to characterize the extent of the expected benefit in terms of specific crops traits and circumstances of the growth season and data collection technology.

### Model II vs. III

For CT and NDVI traits, *H*^2^ estimates were consistently decreased based on variance component estimates from Model III relative to Model II (Table 1), though the magnitude of the difference ranges from 12 to 62%. As for BIC-based model fit comparisons, results proved trait-specific. For CT, Model III showed smaller BIC values, and thus, better fit than Model II. However, for NDVI, most datasets showed better BIC-based fit by Model II compared to Model III (Table 2).

As for GC, the *H*^2^ estimates were increased for all datasets using Model III compared to Model II (Table 1). However, in all cases, BIC indicated a most prevalent better fit of Model II compared to III (Table 2). According to the result, for CT and NDVI traits estimation, considering the image cluster effect introduced in Model III as a factor could improve the phenotypic data quality; however, the row and column effect was still dominate for all the trait estimation in this study.

### Model III vs. IV

In most cases, *H*^2^ estimates obtained using variance component estimates from Model IV were either increased or tied with those computed based on Model III. The one exception was for the CT trait based on the datasets from March 2, 2018 (Table 1). In addition, BIC estimates were smaller for model IV relative to III for all traits and in all cases, thereby indicated consistently improved model fit of Model IV relative to III (Table 2). Recall that Model IV included an additional explanatory variable, namely the camera azimuth angle from the plot center to the camera, could be proved to improve model fit. We observed that recent studies have confirmed that the reflectance observed by UAS are affected by multiple solar angles (Assmann et al., 2018) and camera view angles (Cheng et al., 2019). Compared to previous research, the multispectral images were not collected by the UAS at multiple discrete camera view angles intentionally in this study. Instead, the camera azimuth angles in this study were continuously distributed according to the flight route. Therefore, we considered the camera’s azimuth angle as an explanatory covariate in a more general way and aimed to improve the trait estimation.

In general, based on broad-sense heritability, the highest *H*^2^ estimates for CT and NDVI traits were most often obtained when Model II was used for estimation, whereas for the GC trait, the highest *H*^2^ estimates were obtained from Model IV (Table 1), indicating that the best fitting model may be trait-specific. Specifically, the highest estimates of *H*^2^ for CT and NDVI traits were obtained when row and column effects were recognized in the modeling exercise (i.e., Model II), while for the GC trait, accounting for multiple images and the camera view angle yielded higher *H*^2^ estimates, as shown by Model IV. Other technical aspects or components of experimental design may also be considered for modeling to further explain leftover noise and enhance genetic signal. Moreover, even within a trait, estimation results were not consistent. For example, for the NDVI trait, the dataset of May 16, 2018 yielded the highest *H*^2^ estimates when fitted with Model I_*a*_. This indicates the need for further research to fine-tune processing of UAS imaging technology for efficient and accurate extraction of phenotypes relevant to crop improvement.

### Cost Comparison Between the Two Image Processing Methods

During the Agisoft photogrammetry processing in this study, there is no computing cost difference between generation a single orthomosaic photo or generation of multiple orthorectified images. The export of multiple orthorectified images takes a longer time, though marginal relative to the entire analysis. As the number of orthorectified images per plot has increased compared to only one orthomosaic photo per plot, the trait extraction will take longer time than extracting trait values from the orthomosaic photo. Fortunately, the plot-boundary shapefile only needs to be generated once during trait extraction from the orthomosaic photo and the same shapefile can be used for trait extraction from multiple orthorectified images. Therefore, the analysis pipeline for orthophotos presented here does increase computational time, though a relatively marginal increase compared to overall pipeline computational requirements and the data collection time.

## Conclusion

In this study, we demonstrated open-source and highly reproducible image processing methods and applied it for processing three crop phenotypes obtained by UAS, namely canopy temperature, canopy NDVI, and early-stage ground coverage, to seek the potential to improve quality of trait extraction from UAS-based remote sensing. We compared plot-level phenotypic traits extracted from the orthomosaic image with those obtained from orthorectified images, and we provided evidence that phenotyping by UAS remote sensing could be improved by extracting observations directly from multiple orthorectified images and through proper statistical models that are used to capture and account for technological sources of variability.

While further research will be needed, this study shows preliminary evidence with important practical implications for plant breeding and genetics. First, we developed image processing pipelines that have the potential to automatically generate the orthomosaic and orthorectified images from aerial images, without any need for manual manipulation. Second, we proposed batch processing pipelines to quantify different types of plot-level phenotypic traits, namely CT, NDVI, and GC. In addition, we illustrate how cropping plot-level images from orthorectified images can highly improve the efficiency to link genotypes to phenotypes. This approach can significantly increase the number of image samples per plot, indicating views of a plot from different angles, and provide huge training datasets for image-based deep learning. Finally, we proposed four statistical linear mixed models to efficiently partition sources of variation in each trait, specifically variation introduced by the UAS technology and accompanying image processing, in addition to experimental design. The models provide breeders multiple options to investigate traits extracted from high-throughput UAS-based imaging. Overall, through this study, it is expected that the future of modern breeding could be further highlighted, where in conjunction with powerful genomics and phenomics tools, UAS remote sensing can accelerate the genetic gains in plant breeding to meet the global demand for food, fiber, and fuel.

## Data Availability Statement

The datasets presented in this study can be found in online repositories. The names of the repository/repositories and accession number(s) can be found below: http://people.beocat.ksu.edu/~xuwang/Data_2019_FPS/.

## Author Contributions

XW and JP conceived and designed the study. XW, DS, and BE conducted UAS flights. XW performed image analysis. PS, NB, XW, JP, and DS contributed to the statistical analysis. RS, SM, and FE provided experimental lines. XW, PS, NB, and JP wrote the manuscript. JP directed the overall project. All authors edited and reviewed the manuscript.

## Conflict of Interest

The authors declare that the research was conducted in the absence of any commercial or financial relationships that could be construed as a potential conflict of interest.

## Footnotes

1 www.flir-vue-pro.com/news

2 https://uavmissionplanner.netlify.app/

3 github.com/xwangksu/bip

4 github.com/micasense/imageprocessing

5 github.com/xwangksu/traitExtraction

6 www.flir.com/products/vue-pro-r

7 http://people.beocat.ksu.edu/~xuwang/Data_2019_FPS/
