# Supplementary material for: Improved Accuracy of High-Throughput Phenotyping From Unmanned Aerial Systems by Extracting Traits Directly From Orthorectified Images
Source: Front Plant Sci. 2020 Oct 21;11:587093. doi: 10.3389/fpls.2020.587093 (PMC7609415; doi:10.3389/fpls.2020.587093)
Supplement: Supplementary file 1 [file Data_Sheet_1.PDF]

Table 1. Field experimental details of the three wheat nurseries.

Plant Type	Spring wheat	Winter wheat	
Planting Date	Nov. 21, 2017	Sep. 19, 2017	Sep. 17, 2018
Location	Norman E. Borlaug Experiment Station, Ciudad Obregon, Sonora, Mexico	Kansas State University Ashland Bottom Farm, Manhattan, Kansas, United States	
	27°22'57.6" N, 109°55'34.7" W	39°7'54.2" N, 96°37'12.6" W	39°7'56.4" N, 96°37'10.1" W
Number of Plots	3600	360	336
Number of Entries	1800 including checks	146 including checks	150 including checks
Field Design	Alpha lattice design, with two plots per entry	Two blocks, check varieties planted more than once per block	
Plot Size	1.7 m × 3.4 m	1.5 m × 2.4 m	

Table 2. UAS flight details of the image acquisition experiments in the three wheat nurseries.

Plant Type	Sprint wheat	Winter wheat	
UAS Platform	DJI Matrice 100		
Imaging Sensor	FLIR VUE Pro R	MicaSense RedEdge-M	
Flight Speed	5 m/s	2 m/s	
Flight Date	03/02/2018, 03/19/2018	04/04/2018, 04/12/2018, 04/19/2018, 04/23/2018, 05/16/2018	10/03/2018, 10/11/2018, 10/21/2018, 11/03/2018
Flight altitude	60 m AGL	20 m AGL	
In-Air Flight Duration	~16 min	~14 min	~11 min
Ground Sample Distance of Orthomosaic	8.20 cm/pixel	1.35 cm/pixel	

Table 3. Numbers of plot-level CT values extracted from the orthomosaic (om) and orthorectified (or) images.

Dates	3/2/2018	3/19/2018
Total num. of CT_om	3600	3600
Total num. of CT_or	127237	107185
Min. num. of CT_or per plot	12	14
Max. num. of CT_or per plot	49	40
Median num. of CT_or per plot	36	30

Table 4. Numbers of plot-level NDVI values extracted from the orthomosaic (om) and orthorectified (or) images.

Dates	4/4/2018	4/12/2018	4/19/2018	4/23/2018	5/16/2018
Total num. of NDVI_om	719	719	719	719	719
Total num. of NDVI_or	6353	7780	7173	6890	7093
Min. num. of NDVI_or per plot	5	6	5	6	3
Max. num. of NDVI_or per plot	14	17	16	14	20
Median num. of NDVI_or per plot	9	11	10	10	9

Table 5. Numbers of plot-level GC values extracted from the orthomosaic (om) and orthorectified (or) images.

Dates	10/3/2018	10/11/2018	10/21/2018	11/3/2018
Total num. of GC_om	671	671	671	671
Total num. of GC_or	7253	5202	6053	5169
Min. num. of GC_or per plot	5	3	4	4
Max. num. of GC_or per plot	17	12	14	13
Median num. of GC_or per plot	11	8	9	7
